# Extension of *Drosophila* Lifespan by *Astragalus polysaccharide* through a Mechanism Dependent on Antioxidant and Insulin/IGF-1 Signaling

**DOI:** 10.1155/2021/6686748

**Published:** 2021-02-24

**Authors:** Fujia Yang, Minghui Xiu, Shipei Yang, Xu Li, Wenjuan Tuo, Yun Su, Jianzheng He, Yongqi Liu

**Affiliations:** ^1^Provincial-level Key Laboratory for Molecular Medicine of Major Diseases and The Prevention and Treatment with Traditional Chinese Medicine Research in Gansu Colleges and University, Gansu University of Chinese Medicine, Lanzhou 730000, China; ^2^College of Public Health, Gansu University of Chinese Medicine, Lanzhou 730000, China; ^3^College of Basic Medicine, Gansu University of Chinese Medicine, Lanzhou 730000, China; ^4^Key Laboratory for Transfer of Dunhuang Medicine at the Provincial and Ministerial Level, Gansu University of Traditional Chinese Medicine, Lanzhou, China

## Abstract

Historical literature and pharmacological studies demonstrate that *Astragalus polysaccharide* (APS) has anti-inflammatory and antioxidative effects. Studies into the longevity effects of APS are limited, and the molecular mechanism of lifespan extension by APS is not elucidated yet. Here, the longevity effect of APS was investigated in *Drosophila melanogaster* by feeding dose-dependent APS. APS significantly extended the lifespan and improved the reproduction. Meanwhile, APS increased locomotion, TAG level, and starvation resistance and reduced the mortality rate induced by hydrogen peroxide. The activities of superoxide dismutase (SOD) and catalase (CAT) were increased in flies treated with APS diet. Moreover, APS significantly enhanced expressions of antioxidant genes (*Sod1*, *Sod2*, and *Cat*), *dFoxO*, and 4*E* − *BP*, decreased the expressions of insulin-like peptides (*dilp2, dilp3*, and *dilp5)*, and longevity gene *MTH*. Together, these results indicate that APS can prolong the lifespan by regulating antioxidant ability and insulin/IGF-1 signaling and also enhance the reproduction ability in *Drosophila*. APS may be explored as a novel agent for slowing the aging process and improving reproduction.

## 1. Introduction

Aging is the most basic life characteristic of an organism. It results from the impact of the accumulation of various molecular and cellular damages, leading to functional decline in physical and mental capacity over time [1]. With the accelerated aging society, we have to face the aging-related diseases, such as Alzheimer's disease (AD), Parkinson's disease (PD), cardiovascular diseases, and diabetes [2]. Therefore, research on aging has gained global prominence in recent years and enhanced focus garnered on dietary interventions to combat aging.

Multiple theories of aging have been proposed. The oxidative stress theory indicates that oxidative damage increases with age in many organisms. Many reactive oxygen species (ROS) are produced endogenously and exogenously as mitochondrial dysfunction due to oxidative stress, leading to aging [3]. ROS can destroy biological molecules, such as proteins, lipids, and nucleic acids and cause specific mutations in mitochondrial DNA [4]. To eliminate the accumulation of oxidation products, organisms have two antioxidant defense systems. One is endogenous antioxidants, including superoxide dismutase (SOD), glutathione peroxidase (GPx), and catalase (CAT), which can remove ROS in cells as a first defense line. The other one is exogenous antioxidants, such as vitamin C, carotenoids, and melatonin, which can block the ROS reaction process, limit the production of oxidative free radicals, and slow down the second line of defense in the aging process [5]. The insulin/insulin-like growth factor (IGF)-1 signaling (IIS) pathway is also important to regulate aging in many organisms [6, 7]. It is an evolutionarily conserved intracellular signaling pathway that mediates cell proliferation and the rate of development [8]. Reductions of the IIS pathway provide a robust increase in lifespan [7]. In *Drosophila melanogaster*, the activation of dInR increases a cascade of intracellular phosphorylation, leading to the phosphorylation of dFOXO protein [9, 10]. Reduced IIS causes the translocation of dFOXO from the cytoplasm to the nucleus, which can promote longevity and stress resistance [10].

*Astragalus membranaceus* (Huangqi) is one of the most important Chinese herbs that have been commonly used to treat various diseases or marketed as life-prolonging extracts for more than 2000 years [11]. *Astragalus polysaccharide* (APS), a polysaccharide component, is a major bioactive component of *Astragalus membranaceus* and has various pharmacological actions on anticancer, anti-inflammatory, antiradiation, and immunoregulatory effects [12–14]. During the past few years, many studies have demonstrated that APS has antioxidant and antiaging activity [15–18]. It can increase the total antioxidant capacity and glutathione level and reduce the production of malondialdehyde in vitro and in vivo [15, 16, 19]. APS inhibits mitochondrial permeability transition, prevents mitochondrial oxidative damage, and improves the activity of antioxidant enzymes in the mouse liver and brain mitochondria [19]. APS can also effectively relieve diabetes via improving glucose homeostasis and increasing insulin sensitivity in vitro and in vivo [20, 21]. However, the effects of APS on reproduction and aging have not been deeply studied so far.

In the present study, we selected the fruit flies *Drosophila melanogaster* model to investigate the effects of dose-dependent APS on aging and reproduction. *Drosophila* is a robust animal model with many advantages because of its short lifecycle, low cost, and multiple transgenic strains [22]. The role of APS on the antioxidant ability and IIS signaling was evaluated to dissect the mechanism of APS-extend lifespan.

## 2. Materials and Methods

### 2.1. *Drosophila* Strain and Maintenance

*Drosophilaw* ^1118^ (stock #5905) was obtained from the Bloomington *Drosophila* Stock Center. Flies were reared on a standard cornmeal-molasses medium at 25°C and approximately 60% humidity under a 12 h light/12 h dark cycle. *Astragalus polysaccharides* (APS, Shanghai Yuanye Biotechnology Co., Ltd., Shanghai, China) were added to the food to achieve final concentrations of 0, 0.3, 1, or 3 mg/ml for further experiments.

### 2.2. Lifespan Assay

Newly enclosed flies were separated by sex and randomly divided into four groups (200 flies per group, 20 flies per vial). The control group was fed with standard food, and the other three groups were raised on the food with 0.3, 1, or 3 mg/ml APS individually. All groups were transferred to a fresh medium every 3 days, and mortality was recorded until all flies died. The lifespan curve, median lifespan, and maximal lifespan were obtained and calculated.

### 2.3. Development and Reproduction Assay

Twenty mated female flies were kept in a grape juice medium to lay eggs for 24 h. Sixty eggs per tube were transferred to the tubes containing standard food or food with 0.3, 1, and 3 mg/ml APS, individually. The time that larvae become pupa and the number of pupae in each tube were recorded to assay the role of APS in development.

To measure the effect of APS in reproduction, male or virgin female flies were divided into two groups individually. One group was fed the standard food, while the other group was fed the food containing APS. After 7 days, males and virgin females were mated for 12 h in four different conditions, including C1, males and virgin females without APS training; C2, males with APS training and virgin females without; C3, males without APS training and virgin females with; and C4, males and virgin females with APS training. Then, flies were transferred to a standard medium for 24 h, and the total number of eggs was recorded. Sixty eggs per tube in different conditions were transferred to a standard medium. The developmental time and number of pupae were recorded.

### 2.4. Stress Resistance Assay

Flies were cultured in basic mediums or APS mediums for 25 days and transferred to empty vials for 2 h. For oxidative stress resistance, flies were transferred to new vials containing filter paper that soak 5% H_2_O_2_ and 5% glucose solution in the bottom. For starvation resistance, flies were moved to the vials with 1% agar in the bottom. Dead flies were counted every 4–8 h. The mean survival time was calculated.

### 2.5. Food Intake Detection

Flies were starved in empty vials for 6 h after 25 days feeding in basic mediums or APS mediums. Subsequently, 10 flies per group were transferred to the medium including 5% sucrose, 5% yeast extract, 2% bromophenol blue dye, and 1% agar for 4 h. The amount of blue dye ingested was measured by visual inspections. The abdominal blue grade was detected from 0 (colorless abdomen), 1 (less than 1/3 the length of the abdomen), and 2 (between 1/3 and 2/3 the length of the abdomen) to 3 (more than 2/3 the length of the abdomen). The average feeding score was calculated by averaging the feeding scores of each animal in the vial.

### 2.6. TAG Level Measure

The level of triacylglycerol (TAG) was measured and quantified as previously described [23]. After 25 days APS supplementation, 15 flies were homogenized in 300 *μl* of PBS + 1% Triton-X and immediately heated for 10 mins at 70°C to inactivate lipases. Homogenates were incubated with the same amount of triglyceride reagent (Sigma; T2449) or PBST at 37°C for 45 min. 30 *μl* of each sample was added to 100 *μl* free glycerol reagent (Sigma; F6428) and incubated for 5 min at 37°C. Samples were assayed using a multimode microplate reader at 540 nm. TAG concentration was determined by subtracting the absorbance for the free glycerol in the untreated samples from the total glycerol concentration in samples that have been incubated with triglyceride reagent. The TAG level was calculated based on the triolein-equivalent standard curve. TAG measurement was repeated 3 times.

### 2.7. Climbing Assay

Evaluation of locomotor ability was performed using a negative geotaxis test. After cultured in the food with or without APS for 10, 20, and 30 days, respectively, flies were placed in an empty straight tube. The maximum crawling path was controlled within 12 cm. After 10 mins adaption in tubes, flies were tapped down to the bottom every 1 min interval and repeated 5 times. Flies were allowed to climb up the walls of the vials. The number of flies climbing upwards more than 8 cm within 10 s was recorded. The climbing ability is expressed as the percentage of flies that climbed more than 8 cm. Each experimental group was repeated at least eight times.

### 2.8. Antioxidation Assay

Flies pretreated with APS for 15 and 35 days were used to examine antioxidant ability. Briefly, flies were weighed and frozen in liquid nitrogen. The frozen samples were subsequently homogenized in physiological saline and centrifuged at 4°C and 6000 r/min for 15 min. The supernatants were used to test the activities of superoxide dismutase (SOD) and catalase (CAT) and protein content, according to the instructions of the assay kit (Nanjing Jiancheng Bioengineering Institute, Nanjing, China).

### 2.9. Quantitative RT-PCR Analysis

Following treatment of APS for 15 or 35 days, flies were starved for 2 h and stored at −80°C. Total RNA was extracted using the TRIzol reagent (Invitrogen, Carlsbad, CA, USA) and then synthesized into cDNA. The genes of antioxidant and insulin signaling were selected for quantitative RT-PCR analysis. Ribosomal protein 49 (*Rp49*) expression was used as the internal control. The primer sequences were listed in Table 1. The gene expression was calculated using the comparative threshold cycle (*Ct*) method. The levels of gene expression in all groups were expressed as a ratio to the control group value.

### 2.10. Statistical Analysis

Data were expressed as the means ± standard error of mean (S. E. M). Statistical analysis was performed with GraphPad Prism 6 (Version No. 6, GraphPad Software, La Jolla, CA, USA). Statistical significance was established using one-way ANOVA followed by Dunnett's *t*-test except survivorships. Survivorships among groups were compared and tested for significance with a log-rank test. Statistical significance was set to ^*∗*^*p* < 0.05, ^*∗∗*^*p* < 0.01, and ^*∗∗∗*^*p* < 0.001.

## 3. Results

### 3.1. Effect of APS on the Lifespan

**Figure 1:**
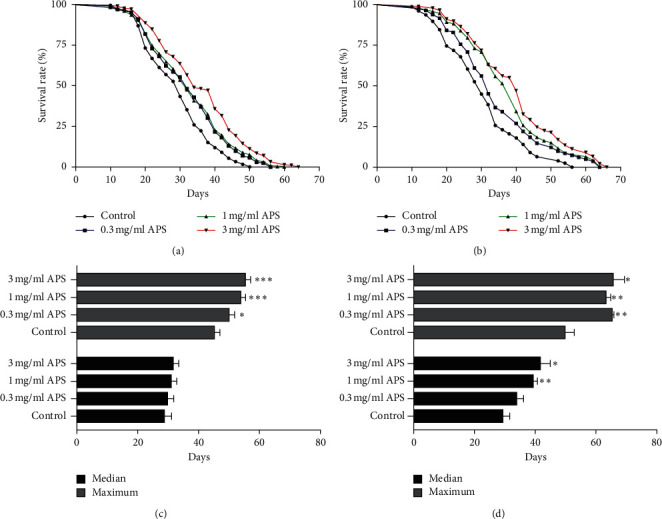
Effect of APS on lifespan in *Drosophila melanogaster*. Survivorship curves in (a) males and (b) females fed diets containing various APS doses. The median and maximal lifespan in (c) males and (d) females. The results are presented as the means ± SEMs (*n* = 12). ^*∗*^*p* < 0.05, ^*∗∗*^*p* < 0.01, and ^*∗∗∗*^*p* < 0.001 indicate significant differences.

To explore the prolongevity effect of APS in fruit flies, the lifespan of wild-type *w*^1118^ flies was measured in the basal media with 0, 0.3, 1, or 3 mg/ml of APS (Figure 1). The lifespan of male and female flies fed APS diet was significantly increased compared to that of control flies (Figures 1(a) and 1(b)). In males, the mean lifespan of flies fed a 3 mg/ml APS diet was obviously increased by 23.41% (*p* < 0.05, Figure 1(a)). APS supplementation at 0.3, 1, and 3 mg/ml in males significantly extended the maximum lifespan. No significant change was observed in the median lifespan of male flies treated with APS (Figure 1(c)). Treatment with APS in female flies resulted in a significant increase in the mean lifespan and maximum lifespan (Figures 1(b) and 1(d)). The high concentration of APS (1 mg/ml and 3 mg/ml) extended the median lifespan of females (Figure 1(d)). Compared with males, it seems that APS has a better effect to prolong the lifespan in females. These results indicate that dose-dependent APS exerts a significant effect on the lifespan, and high concentration of APS remarkably extends the mean lifespan and maximum lifespan.

### 3.2. Effect of APS on Development and Reproduction

**Figure 2:**
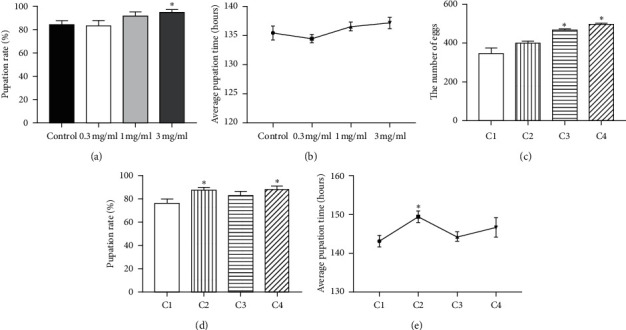
Development and reproduction of flies treated with APS diet. (a) The pupation rate and (b) the average pupation time of flies with development in diet containing APS. (c) Egg numbers, (d) pupation rate, and (e) average pupation time of the offspring of males and virgin females fed diet with or without 3 mg/ml APS for 7 days before mating. C1, males and virgin females without APS training; C2, males with APS training and virgin females without; C3, males without APS training and virgin females with; and C4, males and virgin females with APS training. ^*∗*^*p* < 0.05 represents significant differences.

To define the effect of APS on development, we analyzed the influences of APS on hatchability and growth rate (Figure 2). The hatchability was significantly increased in flies treated with 3 mg/ml APS (*p* < 0.05, Figure 2(a)). However, APS supplementation did not affect the growth rate from an egg to pupa (Figure 2(b)). To further explore the effect of APS on reproduction, we firstly fed males and virgin females with or without 3 mg/ml APS diet for 7 days, followed by cross mating, and then recorded the number of eggs, pupation rate, and growth rate. The number of eggs in females with APS supplementation was significantly increased compared with that in females without APS treatment (*p* < 0.05, Figure 2(c)). The offspring of males with APS supplementation had higher pupation rate than that of males without APS supplementation (*p* < 0.05, Figure 2(d)). In addition, the offspring of ASP supplementation in females without male slightly spent more time to development from an egg to pupa compared with other three groups (Figure 2(e)). These results suggest that APS has a function to enhance the hatchability and oviposition.

### 3.3. Effect of APS on Locomotion and Food Intake

**Figure 3:**
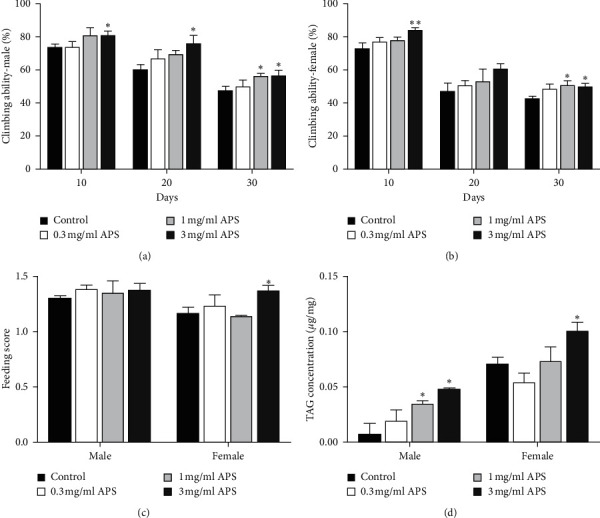
APS supplementation regulated locomotion ability and metabolism. The effect of APS on climbing ability in (a) males and (b) females. (c) Food intake and (d) TAG level of fruit flies fed APS-containing diet or a control diet. ^*∗*^*p* < 0.05 and ^*∗∗*^*p* < 0.01 indicate significant differences.

To prove whether the observed lifespan extending was due to the APS's effects on normal physiology status, the locomotion and food intake of flies fed with APS diet were measured. At 10, 20, and 30 days, 3 mg/ml APS supplementation significantly increased the climbing ability in both males and females (Figures 3(a) and 3(b)). The climbing ability also was increased when flies were fed 1 mg/ml APS diet for 30 days. Dose-dependent APS had no function on food intake in males, while 3 mg/ml APS supplementation slightly increased food intake in females (Figure 3(c)). Then, the TAG levels were measured in both males and females treated with APS for 25 days. 1 mg/ml and 3 mg/ml APS supplementation remarkably increased the body TAG contents in males, while 3 mg/ml APS enhanced the TAG level in females (Figure 3(d)). These data suggest that APS can improve the locomotor ability and regulate lipid metabolism.

### 3.4. Effect of APS on Stress Resistance

**Figure 4:**
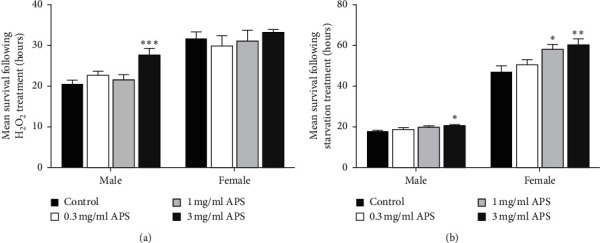
APS supplementation increased the stress resistance in fruit flies. (a) Effect of hydrogen peroxide treatments on survival time in male and female. (b) Starvation resistance in both male and female after 25 days APS supplementation.

To investigate the efficacy of APS on stress resistance, we carried out oxidative stress resistance induced by hydrogen peroxide and starvation resistance. After 25 days dose-dependent APS supplementation, flies were exposed to test the stress resistance. The oxidative stress resistance was visibly increased when males were fed with 3 mg/ml APS diet (*p* < 0.001, Figure 4(a)). However, APS supplementation did not affect the oxidative stress resistance in females. The survival time of males fed 3 mg/ml APS and females fed 1 mg/ml or 3 mg/ml APS was significantly extended following starvation stress (Figure 4(b)). These results indicate that APS can increase resistance to oxidative stress and starvation.

### 3.5. Effect of APS on the Antioxidant Activity of *Drosophila*

**Figure 5:**
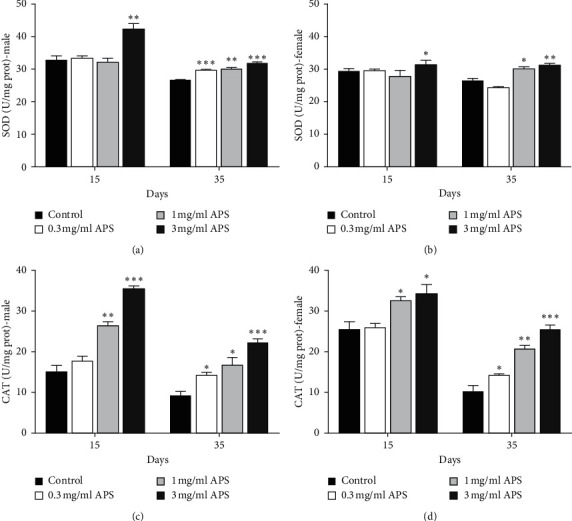
APS supplementation increased the activity of antioxidant enzymes. The activity of SOD in (a) male and (b) female, and the CAT activity in (c) male and (d) female after 15 and 35 days APS supplementation. ^*∗*^*p* < 0.05, ^*∗∗*^*p* < 0.01, and ^*∗∗∗*^*p* < 0.001 show significant differences.

To determine whether APS prolongs the lifespan by enhancing the antioxidant activity of fruit flies, we assayed the effects of APS on the activities of antioxidant enzymes SOD and CAT in flies treated with APS diet for 15 days and 35 days (Figures 5(a) and 5(b)). SOD activity was significantly increased when male and female flies were fed with 3 mg/ml APS for 15 days and 1 mg/ml and 3 mg/ml APS for 35 days (Figures 5(a) and 5(b)). Males treated with 0.3 mg/ml APS for 35 days also had higher activity of SOD than control males (Figure 5(b)). CAT activity was increased when both sex flies were fed 1 and 3 mg/ml ASP diet for 15 and 35 days (Figures 5(c) and 5(d)). 0.3 mg/ml APS supplementation also enhanced the activity of CAT in 35-day-old males and females.

### 3.6. Expression of Antioxidant-Related Genes in *Drosophila* Fed with APS

**Figure 6:**
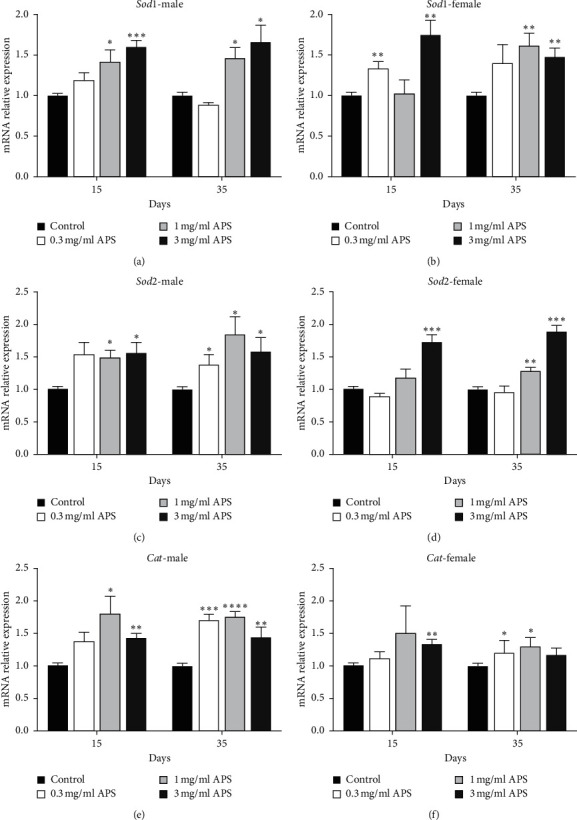
Gene expression analysis of antioxidant genes in fruit flies fed APS diet. Gene expression of *Sod1* in (a) male and (b) female, *Sod2* in (c) male and (d) female, and *Cat* in (e) male and (f) female.

To explore the molecular mechanism of APS prolonging the lifespan of *Drosophila*, we measured the expression of antioxidant-related genes (*Sod1*, *Sod2*, and *Cat*) in flies treated with APS diet for 15 days and 35 days. The mRNA relative expression of genes induced by APS is shown in Figure 6. The mRNA levels of antioxidant enzyme genes *Sod1* and *Sod2* were all significantly upregulated when male and female flies fed with 1 mg/ml and 3 mg/ml APS diet (Figures 6(a)–6(d)). The mRNA expression of *Cat* also increased in flies fed with APS diet for 15 and 35 days (Figures 6(e) and 6(f)). Therefore, APS can promote the expression of antioxidant genes, combined with the increased activity of antioxidant enzymes, and we conclude that the longevity effect of APS may be related to the antioxidant system.

### 3.7. Effects of APS on the Insulin/IGF-1 Signaling

**Figure 7:**
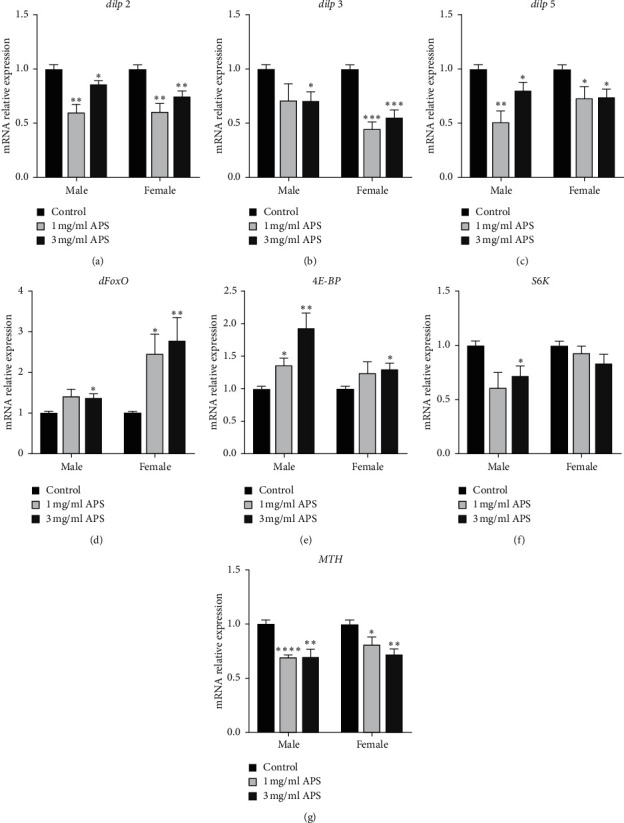
Gene expression analysis of IIS and mTOR signaling genes in fruit flies fed APS diet for 35 days. Effect of APS on relative mRNA expression of (a) *dilp2*, (b) *dilp3*, (c) *dilp5*, (d) *dFoxO*, (e) 4*E* − *BP*, (f) *S6k*, and (g) *MTH* in male and female.

To study whether APS prolongs the lifespan by adjusting the IIS pathway and mTOR pathway that significantly regulate lifespan, we measured the mRNA level of insulin-like peptides (*dilps*) and transcription factor *dFoxO* in the IIS pathway and *S*6*K* and 4*E* − *BP* in the mTOR signaling pathway in flies fed with 1 and 3 mg/ml APS diet for 35 days. The mRNA levels of *dilp2, dilp3*, and *dilp5* were markedly decreased in males and females (Figures 7(a)–7(c)), whereas the mRNA levels of *dFoxO* were significantly increased after 35 days APS supplementation (Figure 7(d)). 3 mg/ml APS supplementation enhanced the expression of 4*E* − *BP* in both males and females (Figure 7(e)) and just inhibited the mRNA level of *S*6*K* in males without in females (Figure 7(f)). In addition, the expression of the Methuselah (*mth*) gene was significantly downregulated in both males and females (Figure 7(g)). The *mth* gene negatively regulates the longevity and stress resistance in fruit flies [24]. These results declare that APS has a function to negatively regulate the IIS pathway and mTOR pathway, and the longevity induced by APS may due to the IIS pathway.

## 4. Discussion

*Astragalus polysaccharide* (APS), one of the major active components of *Astragalus,* has been actively investigated for its nutraceutical benefits including antitumor, antidiabetes, and boosting the immune system. In this study, we aimed to evaluate the effects of APS on reproduction and aging using a *Drosophila* model and found that APS supplementation enhanced the reproduction and development and prolonged the lifespan in both males and females.

This study demonstrated that dose-dependent APS could extend the lifespan in male and female flies. The longevity of APS was observed in silkworm and *C. elegans* as well [18]. However, APS only extended the lifespan in the female silkworm, without males [25]. It may be due to the difference of species. APS has stronger function to regulate aging in females than males, as we found that 1 and 3 mg/ml APS significantly extend median lifespan in females, but it did not affect males. In addition, we found APS supplement can enhance the hatchability rate and promote the growth rate during the development. When virgin females were supplied 3 mg/ml APS for 7 days before mating, the reproductive ability was significantly increased. Previous studies have shown that APS relieves reproductive toxicity in phenobarbital-treated epileptic rats by regulating the reproductive hormones [26]. Administration of live Newcastle disease vaccine with APS in *ovo* significantly improved the development of chicks and enhanced hatchability and gaining weight [27]. Thus, APS can prolong the lifespan and enhance hatchability and reproduction in *Drosophila*.

The effect of APS on lipid metabolism was assessed in this study. The survival under starvation stress conditions was significantly increased in male and female flies after 20 days 3 mg/ml APS supplementation, which may be due to APS affecting the lipid metabolism. We found that 3 mg/ml APS supplementation remarkably increased the TAG level in flies, but it did not significantly affect the food consumption. Previous research has showed that APS can inhibit lipid metabolism via miR-138-5p/SIRT1/SREBP1 pathways in prostate cancer [28] and improve lipid metabolism disorders in diabetic hamsters [29]. In silkworm, APS did not affect food intake [25], which was consistent with our results. Compared to females, males showed the stronger effect of APS on the improved starvation resistance and TAG accumulation. The level of TAG is sex-specifically controlled by several hormones such as insulin, ecdysone, juvenile hormone, and various genes [30]. The mechanism that APS sex-specifically regulates starvation resistance and TAG accumulation needs to be further detected in future.

In our study, the antioxidant activity of APS in flies was proved. Firstly, APS supplementation increased survival under oxidative stress that was treated with H_2_O_2_. Secondly, the activities of SOD and CAT were increased dramatically in both male and female flies treated with APS diet, while the expressions of *Sod1*, *Sod2*, and *Cat* were also increased. These results are consistent with previous reports that APS enhances the activity of SOD and inhibits lactate dehydrogenase (LDH) and malondialdehyde (MDA) in vitro and in mice [15, 19]. In addition, APS can inhibit mitochondrial permeability transition and prevent mitochondrial oxidative damage in the mouse liver and brain mitochondria [19]. Combined with the previous results that APS has a function of antioxidative capacity and immunity in the silkworm [25], we conclude that APS extends lifespan mainly by increasing antioxidative capacity and immunity.

Our results indicated that the longevity benefit of APS was related to the insulin/insulin-like factor signaling (IIS) pathway. APS supplementation increased the expression of *dFOXO* and decreased the expression of insulin-like peptides 2 (*dilp2*), 3 (*dilp3*), and 5 (*dilp5*) in males and females. The transcription factor FOXO is required for growth inhibition and lifespan extension in mammals and *Drosophila* [31]. Activation of FOXO was enough to extend lifespan in flies by decreasing the level of insulin-like peptide 2 and insulin signaling [32]. Loss of *dilp2* was sufficient to extend lifespan [33]. Downregulation of *dilp2* was associated with higher TAG levels and slight resistance to starvation [34]. It is possible that APS increased TAG levels and starvation resistance due to decreased expression of *dilp2* in our studies. Thus, these results indicate that the longevity benefit of APS is mediated via the reduction of IIS signaling. S6K and 4*E* − BP are the downstream effectors of mTOR that negatively regulate lifespan in mammals and *Drosophila* [35]. Reduction of S6K activity can significantly extend the lifespan in fruit flies [36]. Our results showed that ASP supplement slightly decreased the mRNA level of *S6K* in males, without in females. It indicates that APS extending lifespan is not due to mTOR signaling. In addition, we found APS supplement significantly increased the 4*E−BP* level in both males and females. 4*E* − BP is also the transcriptional target of FOXO, which can counteract TORC1 activity on the initiation of cap-dependent mRNA translation in *Drosophila* [37]. FOXO/4*E* − BP can delay age-related muscle weakness at least in part via promoting the activity of the autophagy/lysosome system [38], which can explain why APS enhances the locomotion ability of old flies in our studies. A previous study has showed that APS extends the lifespan of *C. elegans* dependent on the DAF-16/FOXO transcription factor [18]. Therefore, the molecular mechanism of APS-induced lifespan extension is also related with the IIS pathway and autophagy signally.

## 5. Conclusions

**Figure 8:**
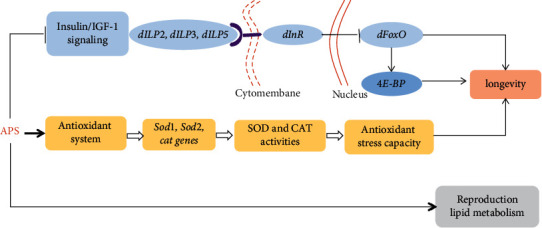
The model indicating the mechanism of APS in extending lifespan and the functions in regulating reproduction and lipid metabolism.

This study demonstrated that diet APS supplementation remarkably prolonged lifespan in both males and females, enhanced the reproduction and development, increased activities of SOD and CAT, and decreased the H_2_O_2_- and starvation-induced mortality rate. The antiaging activity was mostly mediated by the antioxidative capacity and IIS signing pathway (Figure 8). APS may have a potential use in the development of medicines for the treatment of ailments and diseases linked to aging.

## Figures and Tables

**Table 1:** List of forward and reverse used in the gene expression study.

Genes	Forward	Reverse
*Sod1*	GCGGCGTTATTGGCATTG	ACTAACAGACCACAGGCTATG
*Sod2*	CACATCAACCACACCATCTTC	GCTCTTCCACTGCGACTC
*Cat*	TGAACTTCCTGGATGAGATGTC	TCTTGGCGGCACAATACTG
*dFoxO*	AGCAACCTCAGCAACATAAGCAG	TCAGATTTGTGGTAGCCGTTTGTG
*S6k*	CCAAATCATGGGCGAAACC	CTGAACGGCAGATGGTCTGT
4*E* − *BP*	CCAGGAAGGTTGTCATCTCG	CAGGAGTGGTGGAGTAGAGG
*dilp2*	AGCAAGCCTTTGTCCTTCATCTC	ACACCATACTCAGCACCTCGTTG
*dilp3*	TGTGTGTATGGCTTCAACGCAATG	CACTCAACAGTCTTTCCAGCAGGG
*dilp5*	GAGGCACCTTGGGCCTATTC	CATGTGGTGAGATTCGG
*Rp49*	CTTCATCCGCCACCAGTC	GCACCAGGAACTTCTTGAATC

## Data Availability

Data used to support the findings of this study can be obtained from the corresponding author on request.
